# Modulation of oxidative and nitrosative stress attenuates microvascular hyperpermeability in ovine model of *Pseudomonas aeruginosa* sepsis

**DOI:** 10.1038/s41598-021-03320-w

**Published:** 2021-12-14

**Authors:** Satoshi Fukuda, Yosuke Niimi, Yasutaka Hirasawa, Ennert R. Manyeza, C. Edwin Garner, Garry Southan, Andrew L. Salzman, Donald S. Prough, Perenlei Enkhbaatar

**Affiliations:** 1 Department of Anesthesiology, Medical Branch, University of Texas, 301 University Boulevard, Galveston, TX 77555 USA; 2 Department of General Medicine, International University of Health and Welfare, Shioya Hospital, Tochigi, 329-2145 Japan; 3 Department of Plastic and Reconstructive Surgery, Tokyo Women’s Medical University, Tokyo, 162-8666 Japan; 4 Department of Respirology, Graduate School of Medicine, Chiba University, Chiba, 260-8677 Japan; 5 Salzman Group Inc., Beverly, MA 01915 USA

**Keywords:** Translational research, Drug development, Oedema, Infection

## Abstract

In sepsis, microvascular hyperpermeability caused by oxidative/nitrosative stress (O&NS) plays an important role in tissue edema leading to multi-organ dysfunctions and increased mortality. We hypothesized that a novel compound R-107, a modulator of O&NS, effectively ameliorates the severity of microvascular hyperpermeability and preserves multi-organ function in ovine sepsis model. Sepsis was induced in twenty-two adult female Merino sheep by intravenous infusion of *Pseudomonas aeruginosa* (PA) (1 × 10^10^ CFUs). The animals were allocated into: 1) Control (n = 13): intramuscular injection (IM) of saline; and 2) Treatment (n = 9): IM of 50 mg/kg R-107. The treatment was given after the PA injection, and monitored for 24-h. R-107 treatment significantly reduced fluid requirement (15–24 h, *P* < 0.05), net fluid balance (9–24 h, *P* < 0.05), and water content in lung/heart/kidney (*P* = 0.02/0.04/0.01) compared to control. R-107 treatment significantly decreased lung injury score/modified sheep SOFA score at 24-h (*P* = 0.01/0.04), significantly lowered arterial lactate (21–24 h, *P* < 0.05), shed syndecan-1 (3–6 h, *P* < 0.05), interleukin-6 (6–12 h, *P* < 0.05) levels in plasma, and significantly attenuated lung tissue 3-nitrotyrosine and vascular endothelial growth factor-A expressions (*P* = 0.03/0.002) compared to control. There was no adverse effect in R-107 treatment. In conclusion, modulation of O&NS by R-107 reduced hyperpermeability markers and improved multi-organ function.

## Introduction

Sepsis is a life-threatening organ dysfunction caused by a dysregulated host response to infection, and the most frequent cause of death in intensive care units (ICUs) due to multiple organ failure^1,2^. The overall rate of hospital mortality of sepsis is reported at 25–35%^3–5^. Recent work by Luhr et al. reported that mortality rate of septic patients has remained unchanged over the last two decades^6^. Endothelial cell damage and increased microvascular hyperpermeability caused by excessive oxidative/nitrosative stress (O&NS) produce interstitial tissue and multi-organ edema, leading to multiple organ dysfunctions and increased mortality^7–11^.

Various therapies targeting O&NS (i.e., superoxide anion, hydrogen peroxide, nitric oxide, and peroxynitrite) have been proposed for treatment of sepsis, however none of them has advanced to clinical practice as a standard therapy^12–14^. The exact reasons for these failed translational studies are unknown, however, it may be related to the lack of approaches that consider the complexity and multifactorial mechanism of O&NS-induced tissue injury, specifically the imbalance of nitric oxide (NO) and superoxide anion (O_2_^-^) and peroxynitrite in the septic condition^15–20^. The imbalance of these free radical species during sepsis impacts the distribution of extracellular water, disrupts epithelial and endothelial tight junctions, impairs endothelial function and vascular smooth muscle tone, chokes off microcirculatory blood flow, triggers pulmonary arterial hypertension, and raises endothelial hyperpermeability^16–20^.

Previously, we tested the effects of the novel anti-O&NS agent R-100, which has triple actions– O_2_^-^ catalytic degradation, NO donation, and peroxynitrite decomposition catalysis in a clinically-relevant ovine model of pneumonia/sepsis^21^.

In the present study, we further tested the hypothesis that the novel drug R-107, a prodrug of R-100, attenuates microvascular hyperpermeability and improves multi-organ function and survival in an ovine model of *Pseudomonas aeruginosa* (PA) sepsis.

## Results

### Changes in systemic cardiopulmonary hemodynamics and biochemical variables during and after intravenous bacterial infusion

During the 60-min intravenous infusion of PA, body temperature (BT), heart rate (HR), mean arterial pressure (MAP), mean pulmonary artery pressure (mPAP), central venous pressure (CVP), systemic vascular resistance index (SVRI), pulmonary vascular resistance index (PVRI), and respiratory rate (RR) were increased, and cardiac index (CI) was decreased in all animals with the peak at 20–30 min after the start of the PA infusion. These changes returned to the baseline (BL) values by 90 min after the start of the bacterial infusion, with the exception of the BT, HR, mPAP, PVRI, and plasma lactate level, which gradually increased until 180 min after the initiation of the bacterial infusion. The greatest increases of end-tidal CO_2_ (EtCO_2_) and CO_2_ production (VCO_2_) were observed at 30 min; these values then started decreasing 180 min after the start of the bacterial infusion. There was no significant difference in all variables between the two groups, indicating comparable injury in the two groups (Table 1).

**Table 1:** Changes in systemic hemodynamics and biochemical variable during and after (Baseline–180 min) bacterial intravenously administration.

Parameter	Group/Time	BL	30 min	60 min	90 min	120 min	150 min	180 min
Temperature (°C)	Control	39.0 ± 0.1	40.1 ± 0.1	40.2 ± 0.1	40.4 ± 0.1	40.2 ± 0.1	40.5 ± 0.2	40.6 ± 0.2
	R-107	39.1 ± 0.1	40.2 ± 0.2	40.3 ± 0.2	40.1 ± 0.1	39.9 ± 0.2	40.0 ± 0.2	40.2 ± 0.2
Heart rate (beats/min)	Control	92 ± 3	110 ± 8	126 ± 10	163 ± 6	164 ± 6	174 ± 8	161 ± 6
	R-107	97 ± 4	116 ± 9	127 ± 8	171 ± 11	174 ± 13	176 ± 13	162 ± 11
Mean arterial pressure (mmHg)	Control	99 ± 2	116 ± 5	104 ± 3	90 ± 4	93 ± 3	94 ± 5	98 ± 4
	R-107	96 ± 2	110 ± 3	108 ± 7	89 ± 5	90 ± 3	93 ± 3	96 ± 2
Mean pulmonary artery pressure (mmHg)	Control	18 ± 1	43 ± 2	35 ± 1	31 ± 2	31 ± 2	34 ± 2	31 ± 1
	R-107	17 ± 1	43 ± 2	34 ± 2	27 ± 2	27 ± 1	29 ± 1	29 ± 2
Central venous pressure (mmHg)	Control	4 ± 1	8 ± 1	6 ± 1	6 ± 1	7 ± 1	7 ± 1	6 ± 1
	R-107	4 ± 1	7 ± 1	5 ± 1	5 ± 1	6 ± 1	5 ± 1	5 ± 1
Cardiac index (mL/min/m^2^)	Control	6.4 ± 0.3	4.1 ± 0.5	4.6 ± 0.4	6.7 ± 0.4	6.9 ± 0.5	5.8 ± 0.4	5.7 ± 0.5
	R-107	6.3 ± 0.4	3.7 ± 0.4	5.7 ± 0.4	7.1 ± 0.4	6.5 ± 0.4	6.5 ± 0.4	5.4 ± 0.4
Systemic vascular resistance index (dyne-sec/cm^5^/m^2^)	Control	1214 ± 65	2472 ± 2729	1901 ± 199	1037 ± 72	1063 ± 87	1256 ± 100	1449 ± 155
	R-107	1212 ± 85	2427 ± 280	1520 ± 157	973 ± 86	1085 ± 100	1131 ± 102	1418 ± 101
Pulmonary vascular resistance index (dyne-sec/cm^5^/m^2^)	Control	111 ± 10	474 ± 50	320 ± 31	194 ± 24	193 ± 14	255 ± 15	253 ± 24
	R-107	98 ± 9	525 ± 66	269 ± 42	167 ± 20	166 ± 16	190 ± 15	223 ± 29
Respiratory rate (rate/min)	Control	20	25 ± 2	25 ± 2	23 ± 2	23 ± 2	23 ± 2	23 ± 1
	R-107	20	24 ± 2	22 ± 1	23 ± 2	22 ± 2	22 ± 2	22 ± 1
End-tidal CO_2_ (mmHg)	Control	–	32.0 ± 2.9	30.1 ± 2.1	30.7 ± 1.5	32.4 ± 1.1	34.4 ± 2.2	33.1 ± 1.7
	R-107	–	36.3 ± 2.4	31.1 ± 1.6	29.5 ± 1.5	30.2 ± 2.2	33.7 ± 1.9	34.6 ± 2.5
CO_2_ production (VCO_2_) (mL/min)	Control	–	352 ± 33	252 ± 28	264 ± 34	276 ± 22	294 ± 23	290 ± 21
	R-107	–	333 ± 28	249 ± 15	253 ± 13	246 ± 21	284 ± 22	285 ± 23
Lactate (mmol/L)	Control	0.51 ± 0.04	–	2.65 ± 0.29	2.90 ± 0.37	3.00 ± 0.40	–	3.37 ± 0.52
	R-107	0.52 ± 0.05	–	2.20 ± 0.49	2.89 ± 0.60	2.96 ± 0.62	–	2.85 ± 0.61

Data are expressed as mean ± SEM. Two-way analysis of variance with a mixed-effects model with post hoc Bonferroni multiple comparison tests was performed.

### Survival, qSOFA and mSOFA Scores, Plasma Lactate Clearance, and Lung Injury Score

During the 24-h study period, in both groups, MAP was decreased and BT, HR, mPAP, and CI were increased from their BL values, reflecting a state of hyperdynamic sepsis (Table 2). The BT was significantly higher in R-107 treated sheep at 18–21 h (*P* < 0.05) compared to control. R-107 significantly attenuated the increases in LAP and CVP at 21–24 h (*P* < 0.01) compared to control. R-107 treatment also significantly decreased right ventricular stroke work index at 15 h (*P* < 0.05), and significantly attenuated the decreases in static lung compliance at 21 h (*P* < 0.05) as compared to control (Table 2).

**Table 2:** Cardiovascular hemodynamics, pulmonary mechanics, hematocrit, biochemical variables, and systemic neutrophil counts during baseline and at 24-h.

Parameter	Group/time	BL	6 h	12 h	15 h	18 h	21 h	24 h
Temperature (°C)	Control	39.0 ± 0.1	40.8 ± 0.2	40.5 ± 0.2	40.3 ± 0.3	40.1 ± 0.2	40.2 ± 0.3	40.2 ± 0.2
	R-107	39.1 ± 0.1	40.3 ± 0.1	40.6 ± 0.1	40.7 ± 0.2	40.8 ± 0.2 *	40.8 ± 0.2 *	40.8 ± 0.2
Heart rate (beats/min)	Control	92 ± 3	151 ± 8	177 ± 7	181 ± 8	172 ± 5	159 ± 9	164 ± 10
	R-107	97 ± 4	169 ± 8	181 ± 9	184 ± 10	168 ± 11	174 ± 7	163 ± 10
Mean arterial pressure (mmHg)	Control	99 ± 2	98 ± 5	86 ± 4	88 ± 4	86 ± 3	87 ± 5	83 ± 6
	R-107	96 ± 2	104 ± 4	87 ± 3	91 ± 4	93 ± 3	91 ± 4	92 ± 2
Mean pulmonary artery pressure (mmHg)	Control	18 ± 1	31 ± 1	35 ± 2	39 ± 2	38 ± 2	35 ± 2	32 ± 2
	R-107	17 ± 1	31 ± 2	33 ± 2	34 ± 2	35 ± 2	30 ± 2	27 ± 2
Left atrial pressure (mmHg)	Control	6 ± 1	10 ± 1	12 ± 1	15 ± 1	16 ± 1	17 ± 1	17 ± 1
	R-107	6 ± 1	9 ± 1	11 ± 1	11 ± 1	12 ± 1	12 ± 1 **	11 ± 1 ***
Central venous pressure (mmHg)	Control	4 ± 1	8 ± 1	11 ± 1	13 ± 1	13 ± 1	15 ± 1	15 ± 1
	R-107	4 ± 1	8 ± 1	10 ± 1	11 ± 1	10 ± 1	10 ± 1 **	10 ± 1 **
Cardiac index (mL/min/m^2^)	Control	6.4 ± 0.3	4.8 ± 0.4	8.1 ± 0.6	9.5 ± 0.6	9.7 ± 0.8	8.6 ± 0.9	9.3 ± 1.1
	R-107	6.3 ± 0.4	6.2 ± 0.5	8.7 ± 0.5	8.2 ± 0.7	8.2 ± 0.5	8.6 ± 0.7	8.2 ± 0.7
Stroke volume index (mL/m^2^/beat)	Control	71 ± 5	33 ± 4	45 ± 3	53 ± 3	57 ± 5	55 ± 5	57 ± 6
	R-107	66 ± 5	37 ± 3	49 ± 4	46 ± 5	50 ± 3	49 ± 3	51 ± 4
Left ventricular stroke work index (gm-m/m^2^/beat)	Control	86 ± 6	38 ± 6	44 ± 3	50 ± 4	54 ± 5	52 ± 6	54 ± 6
	R-107	78 ± 6	44 ± 4	48 ± 5	48 ± 6	52 ± 3	52 ± 3	54 ± 3
Right ventricular stroke work index (gm-m/m^2^/beat)	Control	13 ± 1	10 ± 1	15 ± 1	19 ± 2	19 ± 2	15 ± 2	13 ± 1
	R-107	11 ± 1	12 ± 1	15 ± 1	13 ± 1 *	17 ± 2	14 ± 1	11 ± 1
Systemic vascular resistance index (dyne-sec/cm^5^/m^2^)	Control	1214 ± 65	1710 ± 262	794 ± 86	672 ± 78	631 ± 48	755 ± 125	700 ± 113
	R-107	1212 ± 85	1303 ± 129	728 ± 54	822 ± 71	834 ± 65	796 ± 73	845 ± 86
Pulmonary vascular resistance index (dyne-sec/cm^5^/m^2^)	Control	111 ± 10	273 ± 30	223 ± 26	188 ± 20	181 ± 32	192 ± 33	155 ± 40
	R-107	98 ± 9	220 ± 30	176 ± 30	202 ± 32	189 ± 14	160 ± 12	131 ± 16
PaO_2_/FiO_2_ ratio (mmHg)	Control	513 ± 6	467 ± 29	402 ± 33	362 ± 35	320 ± 45	274 ± 45	229 ± 45
	R-107	520 ± 9	507 ± 27	433 ± 50	383 ± 63	380 ± 80	363 ± 84	356 ± 85
Shunt fraction (%)	Control	6 ± 1	7 ± 1	15 ± 3	22 ± 4	27 ± 6	31 ± 8	32 ± 5
	R-107	5 ± 1	5 ± 1	15 ± 3	20 ± 5	20 ± 7	19 ± 6	22 ± 8
Peak airway pressure (cmH_2_O)	Control	20 ± 1	26 ± 1	32 ± 2	33 ± 2	33 ± 2	34 ± 2	32 ± 2
	R-107	17 ± 1	26 ± 2	31 ± 3	33 ± 3	29 ± 3	28 ± 3	27 ± 3
Plateau airway pressure (cmH_2_O)	Control	18 ± 1	22 ± 1	30 ± 2	31 ± 2	31 ± 2	32 ± 2	29 ± 2
	R-107	16 ± 1	23 ± 2	28 ± 3	29 ± 3	27 ± 3	25 ± 3	25 ± 3
Lung static compliance (mL/cmH_2_O)	Control	35 ± 2	26 ± 1	17 ± 2	15 ± 2	13 ± 2	14 ± 2	17 ± 2
	R-107	40 ± 3	28 ± 3	22 ± 4	20 ± 4	24 ± 5	26 ± 4 *	24 ± 3
Glucose (mg/dL)	Control	62 ± 2	47 ± 2	51 ± 10	46 ± 4	47 ± 4	46 ± 4	49 ± 5
	R-107	69 ± 2	47 ± 4	48 ± 5	47 ± 2	46 ± 2	44 ± 1	45 ± 2
Neutrophil (× 10^3^ cell/μL)	Control	2.0 ± 0.2	0.6 ± 0.1	2.1 ± 0.4	-	3.6 ± 0.7	-	3.2 ± 1.5
	R-107	2.3 ± 0.3	1.4 ± 0.7	4.0 ± 1.2	-	5.4 ± 1.7	-	5.7 ± 2.0

Data are expressed as mean ± SEM. Two-way analysis of variance with a mixed-effects model with post hoc Bonferroni multiple comparison tests was performed. *P < 0.05, **P < 0.01, and ***P < 0.001.

The 24-h survival rate was 89% (8 out of 9 sheep) for the R-107 treatment group, and 69% (9 out of 13 sheep) for control (no statistical significance).

**Figure 1:**
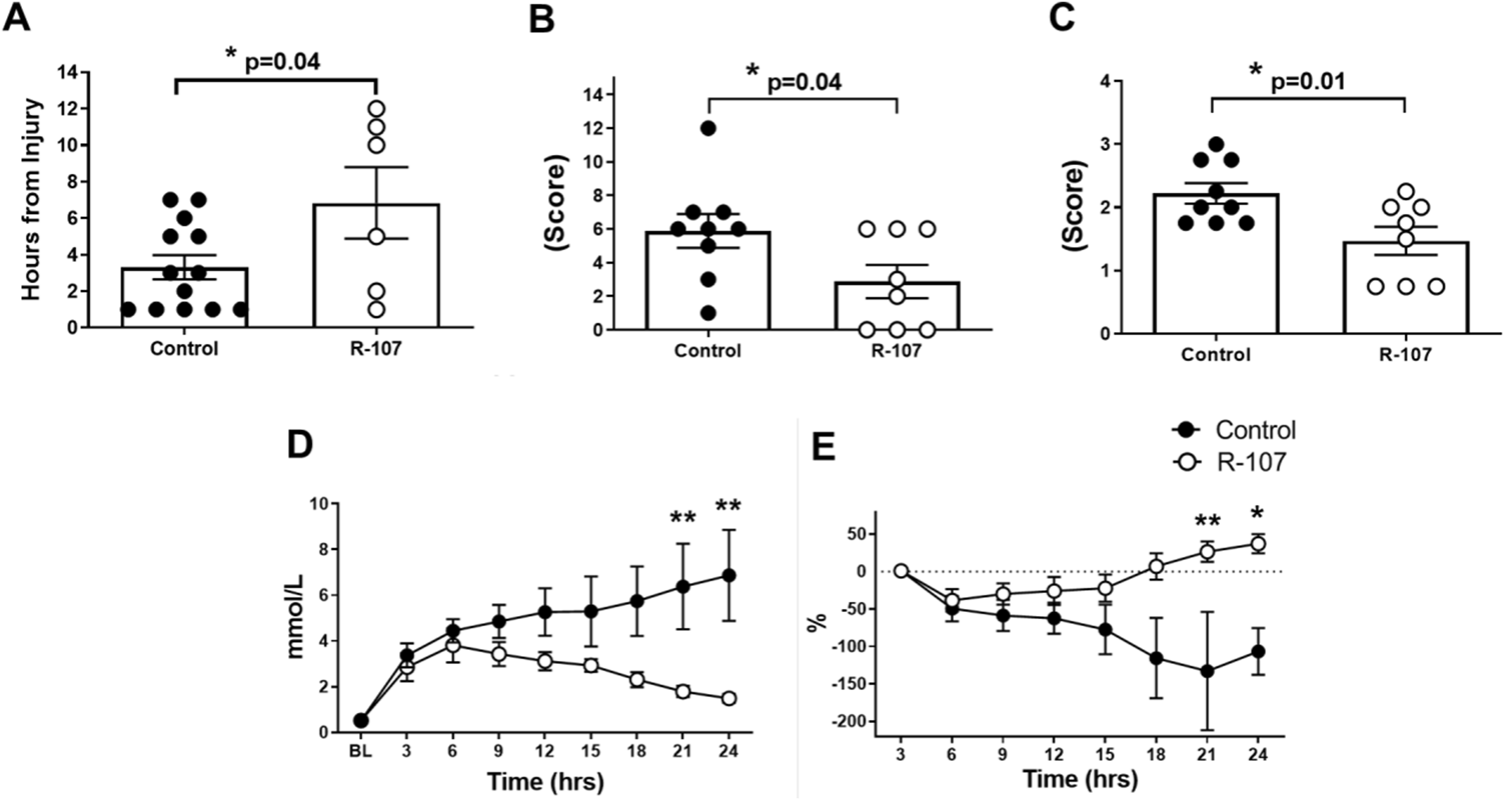
Quick SOFA and modified sheep SOFA scores, plasma lactate clearance, and lung injury score. (**A**) Time met the quick SOFA criteria from the injection of *Pseudomonas aeruginosa* in two groups (n = 13 in control and n = 6 in the R-107 treatment group. The data in three out of nine sheep in the treatment group have not been shown because these sheep did not develop the quick SOFA criteria). (**B**) Modified sheep SOFA scores at 24-h in two groups (n = 9 in control and n = 8 in the R-107 treatment group at 24-h. Four out of thirteen sheep in control and one out of nine sheep in the treatment group expired at 24-h timepoint (the time of SOFA score determination). (**C**) Lung injury score at 24-h in two groups (n = 9 in control and n = 8 in the R-107 treatment group. Four out of thirteen sheep in control and one out of nine sheep in the treatment group expired at 24-h timepoint). (**D**) Plasma lactate concentration during the baseline and at 24-h in two groups. (**E**) Lactate clearance from the 3-h value during the baseline and at 24-h in two groups. Closed circles represent control group and open circles represent the R-107 treatment group. Data are expressed as mean ± SEM. (*P < 0.05, **P < 0.01, and ***P < 0.001, Control vs. R-107 treatment group.).

R-107 treatment significantly delayed the onset of sepsis compared to control, which was confirmed by the time that sheep met quick SOFA (qSOFA) criteria. Six of 9 sheep in the R-107 group met the qSOFA score criteria within 6.8 ± 2.0 h, whereas all 13 sheep in the control group met these criteria within 3.3 ± 0.7 h from the start of the PA infusion (*P* = 0.04) (Fig. 1A).

R-107 treatment significantly attenuated the increases in the modified sheep SOFA (mSOFA) score at 24-h in the survived animals (n = 8 in the R-107 treatment group and n = 9 in control, 2.9 ± 1.0 vs. 5.9 ± 1.0, *P* = 0.04) as compared to control (Fig. 1B). The measured variables of mSOFA score in both groups are shown in Table 3.

**Table 3:** mSOFA Score Variables at 24-h.

Variable of mSOFA score/group		*Respiration* PaO_2_/FiO_2_ (mmHg)	*Coagulation* Platelets (× 10^3^/μL)	*Liver* Bilirubin (mg/dL)	*Cardiovascular* MAP decrement from BL (mmHg)	*Central nervous system* SSNAA (2–11)	*Renal* Creatinine (mg/dL)	Total mSOFA score (at 24-h)
Control	mSOFA Score	2.1 ± 0.5	(0)	(0)	1.2 ± 0.4	2.2 ± 0.4	0.3 ± 0.2	5.9 ± 1.0
	(Variables at 24-h)	(229 ± 44)	(274 ± 39)	(0.5 ± 0.1)	(11.3 ± 5.2)	(6.2 ± 0.9)	(1.0 ± 0.2)	
R-107	mSOFA Score	1.6 ± 0.7	0.4 ± 0.4	(0)	0.3 ± 0.2	0.6 ± 0.3	(0)	2.9 ± 1.0
	(Variables at 24-h)	(356 ± 85)	(341 ± 72)	(0.2 ± 0.1)	(4.5 ± 2.6)	(9.6 ± 0.7)	(0.7 ± 0.1)	

Also, the lung injury score (LIS) at 24-h in the survived animals was significantly lower in the R-107 treatment group as compared to control (n = 8 in the R-107 treatment group and n = 9 in control, 1.5 ± 0.2 vs. 2.2 ± 0.2, *P* = 0.01) (Fig. 1C).

R-107 treatment significantly attenuated the increase in plasma lactate levels at 21–24 h (*P* < 0.01) (Fig. 1D), and significantly improved lactate clearance during sepsis compared to control at 21–24 h (*P* < 0.05) (Fig. 1E).

### Fluid balance, hematocrit changes, plasma protein concentration, and total amount of pleural and ascitic fluid at necropsy

**Figure 2:**
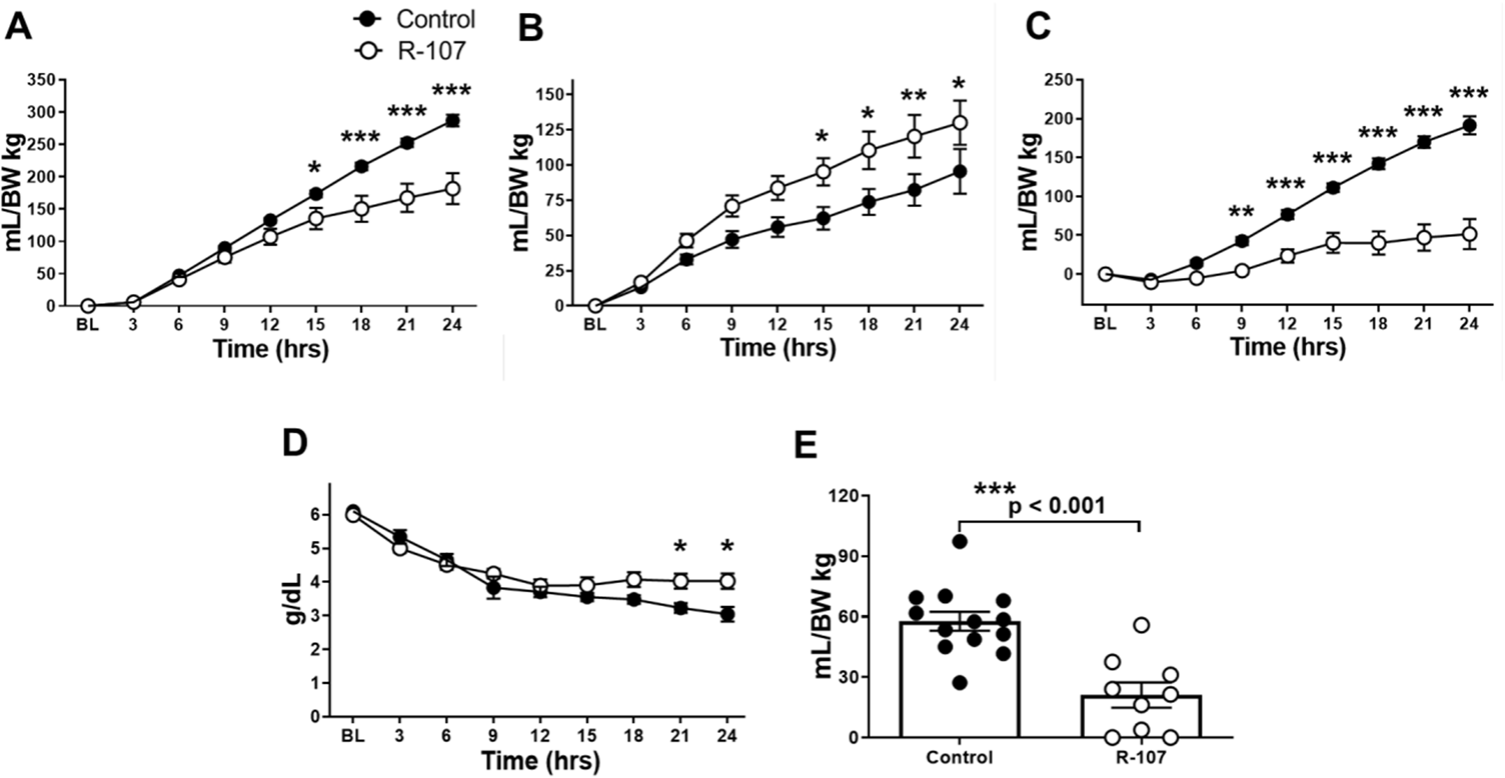
Fluid balance, plasma protein concentration, and total amount of pleural and ascitic fluid at necropsy. (**A**) Fluid requirement during the baseline and at 24-h in two groups. (**B**) Cumulative urine output during the baseline and at 24-h in two groups. (**C**) Net fluid balance during the baseline and at 24-h in two groups. (**D**) Plasma protein concentration during the baseline and at 24-h in two groups. (**E**) Combined volume of pleural and ascitic fluid measured at euthanasia in two groups. Closed circles represent control group and open circles represent the R-107 treatment group. Data are expressed as mean ± SEM. (*P < 0.05, **P < 0.01, and ***P < 0.001, Control vs. R-107 treatment group.).

The fluid requirement was significantly lower in the R-107 treated group at 15–24 h (*P* < 0.05) (Fig. 2A), and cumulative urine output at 15–24 h (*P* < 0.05) (Fig. 2B) was significantly higher in the R-107 treated group compared to control. The net fluid balance was significantly attenuated in the R-107 treated group as compared to control at 9–24 h (*P* < 0.05) (Fig. 2C). The hematocrit levels were comparable between the treated and control groups.

The plasma protein concentration was significantly higher in the R-107 treated group compared to control at 21–24 h (*P* < 0.05) (Fig. 2D).

The combined volume of pleural and ascitic fluid at euthanasia was significantly lower in the R-107 treated group as compared to control (21.1 ± 6.2 vs. 57.7 ± 4.7 mL/kg BW, *P* < 0.001) (Fig. 2E).

### Organ extravascular water content and bacterial clearance

**Figure 3:**
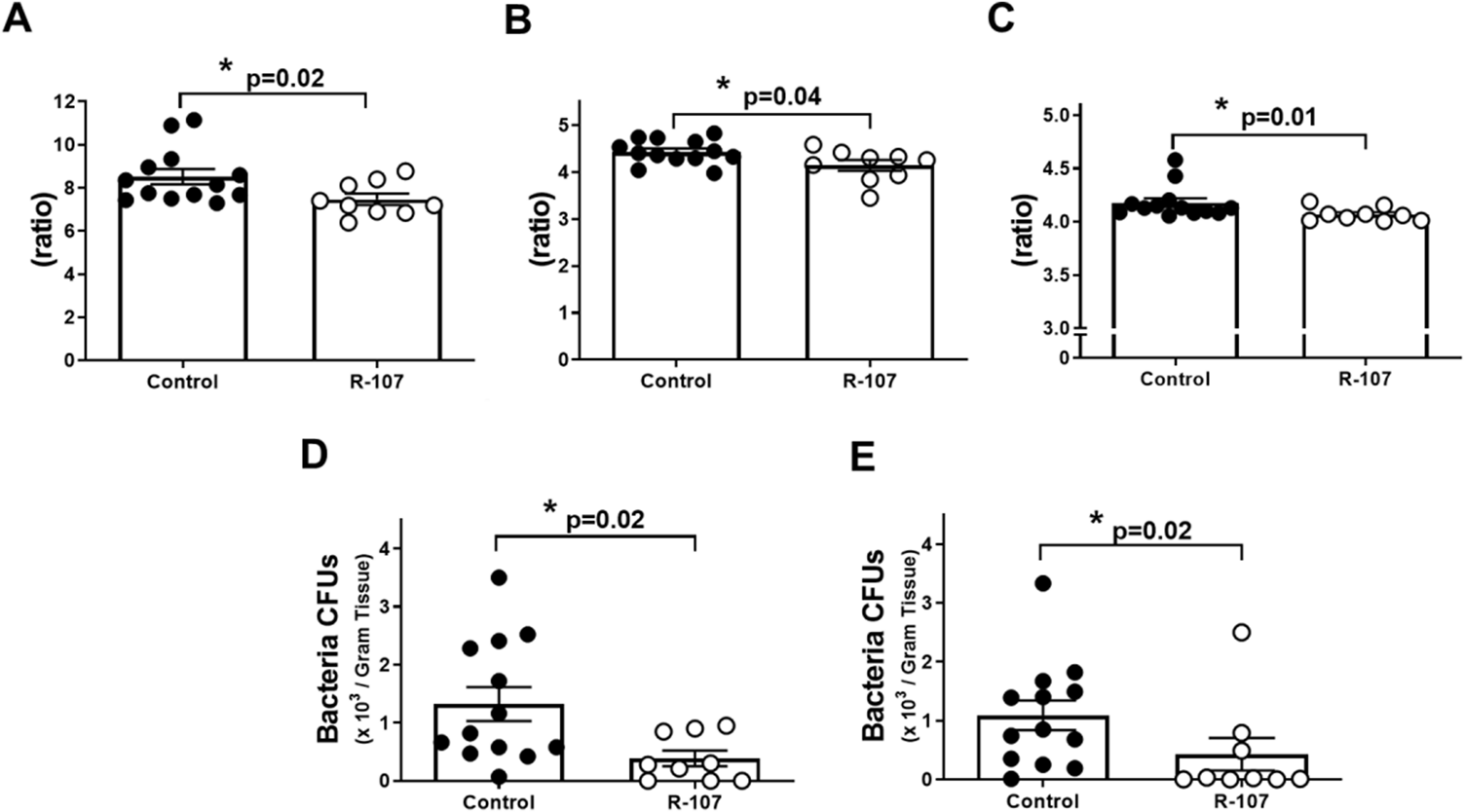
Organ extravascular water content and bacterial clearance. (**A**) Lung extravascular water content at euthanasia in two groups. (**B**) Heart extravascular water content at euthanasia in two groups. (**C**) Kidney extravascular water content at euthanasia in two groups. (**D**) Numbers of bacteria in lung tissue homogenate cultures collected at euthanasia in two groups. (**E**) Numbers of bacteria in kidney tissue homogenate cultures collected at euthanasia in two groups. Closed circles represent control group and open circles represent the R-107 treatment group. Data are expressed as mean ± SEM. (*P < 0.05, **P < 0.01, and ***P < 0.001, Control vs. R-107 treatment group.).

The organ extravascular water content was determined by measuring organ wet-to-dry weight ratio (W/D) at euthanasia. The R-107 treated group displayed significantly lower water content in the lung, heart, and kidney compared to control (lung: 7.5 ± 0.3 vs. 8.5 ± 0.4, heart: 4.1 ± 0.1 vs. 4.4 ± 0.1, and kidney: 4.1 ± 0.02 vs. 4.2 ± 0.04, *P* = 0.02, 0.04, and 0.01, respectively) (Fig. 3A–C).

The number of bacteria in lung and kidney tissue homogenate cultures was significantly lower in the R-107 treated group compared to control (0.4 ± 0.1 vs. 1.3 ± 0.3 and 0.4 ± 0.3 vs. 1.1 ± 0.3 × 10^3^ CFUs/gram tissue in control, *P* = 0.02 and 0.02, respectively) (Fig. 3D,E).

### Oxidative and nitrosative stress, glycocalyx, and inflammatory mediators

**Figure 4:**
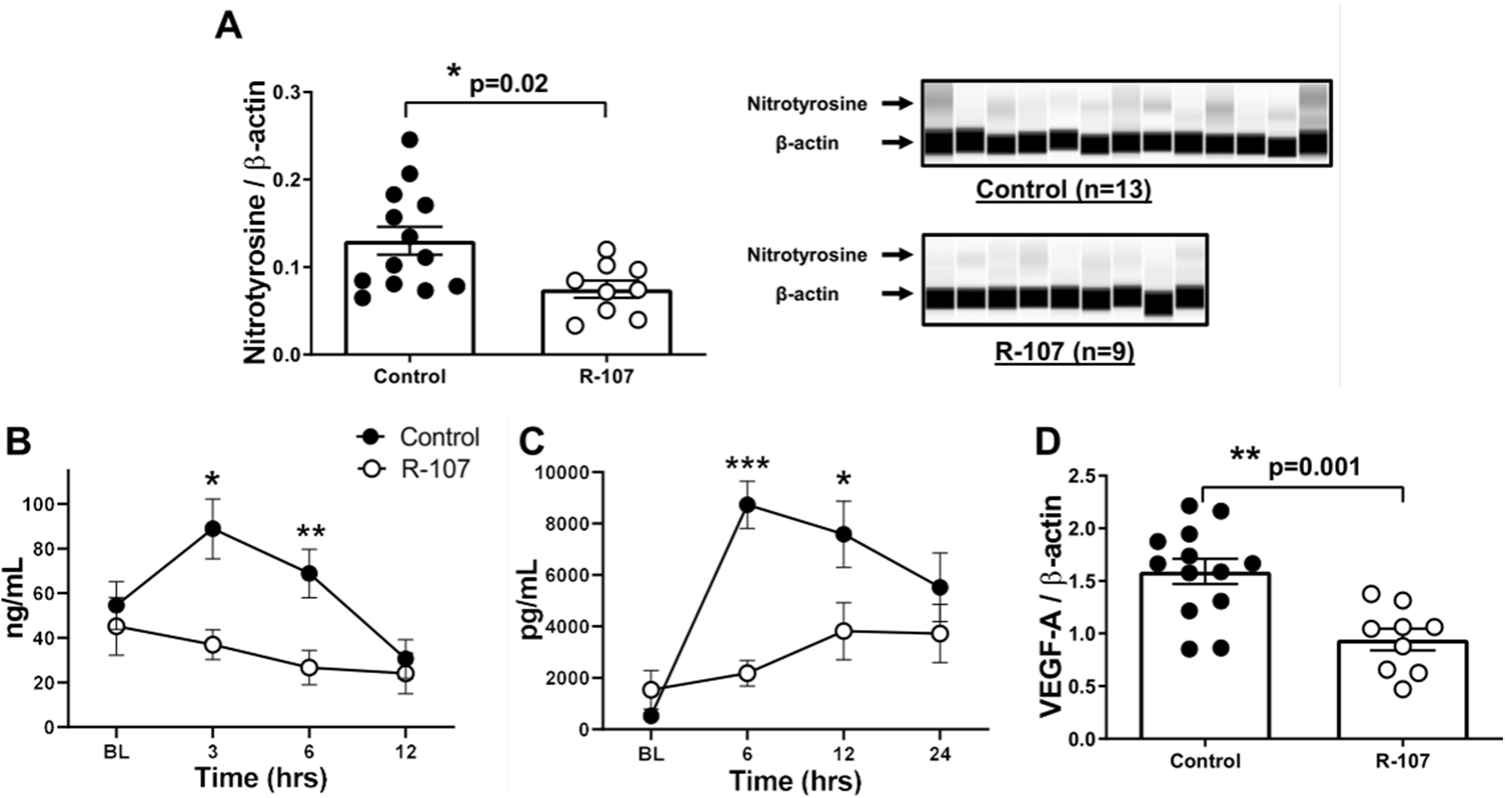
Oxidative and nitrosative stress, glycocalyx, and inflammatory mediators. (**A**) 3-nitrotyrosine level in lung tissue homogenate collected at euthanasia in two groups. (**B**) Plasma shed syndecan-1 concentration during the baseline and at 12-h in two groups. (**C**) Plasma interleukin-6 concentration during the baseline and at 24-h in two groups. (**D**) Vascular endothelial growth factor-A level in lung tissue homogenate collected at euthanasia in two groups. Closed circles represent control group and open circles represent the R-107 treatment group. Data are expressed as mean ± SEM. (*P < 0.05, **P < 0.01, and ***P < 0.001, Control vs. R-107 treatment group.).

The modulation of O&NS by the R-107 treatment was measured by the levels of 3-nitrotyrosine in the lung tissue. The 3-nitrotyrosine levels in lung tissue homogenate collected at the euthanasia was significantly lower in the R-107 treated group compared to control (0.07 ± 0.01 vs. 0.13 ± 0.02, *P* = 0.02) (Fig. 4A).

R-107 significantly decreased plasma levels of shed syndecan-1 (Sdc1) at 3 and 6 h (37.0 ± 6.6 vs. 88.9 ± 13.4 and 26.7 ± 7.7 vs. 68.8 ± 10.9, *P* = 0.005 and 0.03, at 3 and 6 h, respectively) compared to control (Fig. 4B).

The plasma interleukin-6 (IL-6) concentration was significantly lower in the R-107 treated group at 6 and 12 h (2178 ± 467 vs. 8732 ± 918 and 3817 ± 1115 vs. 7585 ± 1287, *P* < 0.001 and 0.03, respectively) compared to control (Fig. 4C). The lung tissue vascular endothelial growth factor-A (VEGF-A) levels were significantly lower in the R-107 treated group compared to control (0.94 ± 0.10 vs. 1.59 ± 0.12, *P* = 0.001) (Fig. 4D).

## Discussion

In this study, we demonstrated that modulation of O&NS by R-107 significantly reduced microvascular hyperpermeability and body fluid retention, resulting in improved multi-organ function and survival in septic sheep. Previously, we demonstrated that the modulation of O&NS by R-100 significantly reduces systemic fluid retention and improves pulmonary gas exchange in the model of ovine pneumonia/sepsis^21^.

The present study results were in an agreement with previous work in terms of reduced fluid retention, organ edema, and microvascular hyperpermeability. The survival rate in control groups in present and previous studies was also comparable (69% vs. 65–70%, respectively^23,37^. In the present study, we further showed that R-107 treatment improved bacterial clearance in the lung and kidney, inhibited increases in inflammatory mediators (plasma shed Sdc1, IL-6, and lung VEGF-A levels), delayed the onset of sepsis (measured by the qSOFA score), attenuated multi-organ dysfunction (measured by the mSOFA score), and improved multi-organ function. In ICU patients with sepsis and septic shock, positive fluid balance, at least in part, due to this microvascular hyperpermeability is not only the independent predictor of mortality, but it also is linked to the increased ICU-days, ventilator-days, and total hospital care costs^38–42^. Therefore, therapies targeting microvascular hyperpermeability and edema formation during sepsis are of particular interest.

As mentioned, sepsis increases microvascular hyperpermeability and excessive fluid retention and causes multiple organ dysfunction by excessive O&NS^7–9,43,44^. Excessive O&NS causes endothelial cell damage and endothelial glycocalyx layer disruption^43–46^. The endothelial glycocalyx layer controls endothelial permeability to water and serves as a barrier to neutrophil/bacteria migration into organ interstitial space from the bloodstream^7–9,44–47^. The Sdc1 is one of the major components of the glycocalyx, and the levels of shed Sdc1 in plasma are considered as a marker of glycocalyx layer disruption during sepsis^48^. Moreover, the plasma shed Sdc1 levels correlate with the increments of plasma IL-6 level during sepsis^49,50^.

In addition, VEGF-A, a well-known inflammatory mediator and potent vascular hyperpermeability factor, is increased in septic conditions^33,35,51^. In our previous studies, we have shown increases in lung tissue VEGF and myeloperoxidase activity associated with sever microvascular hyperpermeability^35^.

The results of the present study demonstrated that R-107 treatment significantly reduced the degree of O&NS as evidenced by increases in lung tissue 3-nitrotyrosine levels. We also showed that R-107 significantly reduced the plasma levels of shed Sdc1 and IL-6 and significantly attenuated VEGF-A levels in lung tissue compared to control. Although, these findings do not represent causation, we speculate that R-107 attenuated microvascular permeability in this model by inhibiting the potent permeability factor VEGF and preserving endothelial glycocalyx through modulation of O&NS stress.

In the present study, we reported that the number of bacteria in lung and kidney tissue were significantly lower in the R-107 treatment group compared to control. The underlying mechanisms for this finding are not fully understood. Lubkin et al. reported that PA secretes toxins that disrupt the endothelial cell monolayer barrier mechanism via the induction of oxidative stress^52,53^. We speculate that protection of the endothelial barrier by R-107 may be attributed to the reduced number of bacteria in organ tissues. Of note, circulating numbers of neutrophils were not affected by R-107, indicating that differences in bacterial numbers between the two groups were not related to variations in the number of neutrophils.

Further, increases in plasma shed Sdc1 in control were unlikely to have been impacted by the volume of resuscitation fluid because the hematocrit, plasma protein concentration, and hemodynamic variables were comparable in both groups at 3–6 h, when the plasma shed Sdc1 levels were significantly lower in the R-107 treated group compared to control.

Our present study has several limitations. First, we did not show causative factors by which R-107 reduced microvascular hyperpermeability, rather we showed co-association of the numbers of potent inflammatory mediators with increased vascular permeability. Second, we did not use the supportive therapies, such as antibiotics and vasopressors, which are the standard therapies for sepsis. However, we aimed to explore a “pure” effects of testing compound without any drug interactions. Third, the study period was relatively short (24-h), which does not consider the concomitant diseases or factors that are associated with human sepsis. However, the model allowed us to most closely mimic the human hyperdynamic sepsis, continuously monitoring cardiopulmonary hemodynamics in a conscious state without the effects of anesthetics. Finally, we have compared variables only in two groups of septic sheep (treated and control) without including the variables obtained from uninjured healthy (Sham) sheep. However, we have repeatedly reported in our previous studies that the preceded surgical procedures in the Sham sheep did not affect variables reflecting multiorgan functions (i.e., cardiopulmonary hemodynamics, pulmonary function) and survival^23,37^.

In conclusion, modulating excessive O&NS stress by R-107 may be considered as an effective and safe therapeutic option for management of sepsis-induced microvascular hyperpermeability. Future studies are warranted to further investigate the mechanisms of how moderation of O&NS by this potential therapeutic compound "R-107" attenuates the pathophysiology of sepsis-induced microvascular hyperpermeability.

## Methods

### Animal model and experimental design

The study was approved by the Institutional Animal Care and Use Committee (IACUC) of the University of Texas Medical Branch and conducted in compliance with the guidelines of the Animal Research: Reporting of In Vivo Experiments^22^, the National Institutes of Health, and the American Physiological Society for the care and use of laboratory animals, as previously described^23,24^.

Twenty-two adult female Merino sheep (body weight [BW] 36.8 ± 1.0 kg) were used. Briefly, animals were anesthetized with an intravenous injection of ketamine and isoflurane inhalation, and multiple vascular catheters were surgically inserted (Swan-Ganz, femoral arterial, and left atrial catheters). Pre- and post-surgical analgesia was provided with long-acting (for 72 h) Buprenorphine SR™ (0.05 mg/kg, SR Veterinary Technologies, Windsor, CO). Merino sheep were chosen because of their close resemblance of the pathophysiologic and immune responses to infection that are seen in humans^25–27^.

After 5–7 days following instrumentation, BL cardiopulmonary hemodynamic variables were collected (Table 2), as previously described^23,24^. After the BL data were collected, a tracheostomy tube and urine catheter were inserted under ketamine and inhaled isoflurane anesthesia, and animals were placed on a mechanical ventilator (AVEA; Carefusion, Yorba Linda, CA) with the initial settings of a pressure-regulated volume control assist-control mode, tidal volume (TV) of 12 mL/kg, positive end-expiratory pressure of 5 cmH_2_O, RR of 20 breaths/minute, and inspired oxygen concentration (FiO_2_) of 0.21. Then, 1.0 × 10^10^ CFUs of *Pseudomonas aeruginosa* (strain; PD-05144 [12-4-4, BRK-1244, NCIB-10780, NRRL-B-3224], catalog #: ATCC® 27,317™, ATCC, Manassas, VA) suspended in 50 mL of warm 0.9% sodium chloride were intravenously injected (IV) via the jugular vein over 60 min in a conscious state. The variables of systemic hemodynamics were continuously monitored during and until 180 min after the initiation of the bacterial IV infusion. (Table 1). Arterial lactate levels were also determined during this time period.

### Animal grouping, drug treatment, and post-injury care

After the injury, animals were randomly allocated into two groups: (1) control: treated with an intramuscular injection (IM) of saline, n = 13; and 2) R-107: administered with IM 50 mg/kg R-107, n = 9. The R-107 was injected into the animal’s right quadriceps immediately after completing the infusion of bacteria.

Briefly, R-107 has a multi-functional prodrug technology to target redox imbalance of O&NS. R-107 is a prodrug ester that hydrolyzes to form R-100^21^, a molecule serving as: a NO donor via its organic nitrate, and a broad-spectrum catalyst of O&NS degradation via its nitroxide moiety (hydroxymethylproxyl). The spectrum of O&NS degradation of R-100 includes: O_2_^-^ dismutation, catalase-like activity (detoxifying H_2_O_2_), and peroxynitrite decomposition. There are at present no approved agents with this multi-functional action. R-107 was provided from the Salzman Group Inc. (Beverly, MA).

Cardiopulmonary hemodynamic variables were continuously monitored (IntelliVue MP50; Philips Medical Systems, Andover, MA) (Table 2), and recorded hourly for a 24-h study period in mechanically-ventilated conscious sheep. RR and FiO_2_ were adjusted to maintain PaCO_2_ between 30—40 mmHg and PaO_2_ ~ 100 mmHg, respectively. Arterial and venous blood gas (i.e., arterial and venous PO_2_, PCO_2_, saturation, lactate, hematocrit) were determined using a blood gas analyzer (RAPIDPoint 500; Siemens Healthcare, Erlangen, Germany). Lactate clearance^28^ was calculated using the following formula: lactate clearance = (lactate _3-h value_-lactate _delayed time-point value_) / lactate _3-h value_ × 100 (expressed as percentage). Plasma protein concentration was measured using a handheld refractometer (National Instrument Company Inc., Baltimore, MD).

Animals were fluid resuscitated with lactated Ringer’s solution (LR; Baxter Healthcare Corporation, Deerfield, IL), starting with an initial infusion rate of 2 mL/kg/hr for 3 h. Thereafter, the LR rate was adjusted every 3 h to maintain hematocrit close to the BL levels ± 3%. Fluid input and urine output were monitored hourly and cumulative fluid balance was calculated, as previously described^23,24^.

### Quick SOFA and Modified Sheep SOFA Score

In order to assess the onset of sepsis and the severity of multi-organ dysfunction during sepsis, we used qSOFA and mSOFA scores, as previously described^1,24,29^. We measured the time to meet the qSOFA criteria from the initiation of the PA injury. Also, the mSOFA score included the values of animal neurological status, MAP, PaO_2_/FiO_2_ ratio, total platelet counts measured by ADVIA-120 (Siemens Healthcare Diagnostics, Deerfield, IL), and plasma total bilirubin and creatinine concentrations measured by the hospital clinical chemistry laboratory. The neurological status of animal was assessed by the Simplified Sheep Neurological/Alertness Assessment score^29^. The qSOFA score was measured hourly until the animals met the criteria, and the mSOFA score was measured at BL and at the 24-h timepoint.

### Euthanasia, tissue collection, and tissue extravascular water content analysis

After completion of the 24-h study period, animals were euthanized with injection of ketamine (40 mg/kg), buprenorphine (0.01 mg/kg), and xylazine (3.0 mg/kg), following the IACUC approved protocol and American Veterinary Medical Association Guidelines for Euthanasia^30^. Immediately after euthanasia, organs and tissues were collected, and lung, heart, and kidney tissue water content were measured by W/D, as previously described^23,24,31^.

### Bacterial clearance in lung and kidney

To assess bacterial clearance in the lung and kidney, a 1.0-g section of the dorsal edge of right lung middle lobe and the kidney cortex were taken at necropsy and homogenized in 2 mL of 1 × phosphate-buffered saline, using a Bullet Blender (Next Advance, Averill Park, NY) following the device protocols (https://www.nextadvance.com/homogenizer-tissue-cell-culture-bullet-blender-support/homogenizer-cell-disrupter-protocols/). Then, 200 µL of the tissue homogenates were transferred onto soy agar plates. The plates were incubated for 24 h at 37 °C for bacterial CFUs counts, as previously described^29,32^.

### Western blotting and enzyme-linked immunosorbent assay

Levels of lung tissue 3-nitrotyrosine (06–284; MilliporeSigma, MA) and VEGF-A (ab46154; Abcam, MA), which is a major mediator of microvascular hyperpermeability^33–35^, were determined by automated capillary Western blot analysis (Wes™ ; ProteinSimple, San Jose, CA), as previously described^24,36^.

The plasma Sdc1 and IL-6 levels were measured by enzyme-linked immunosorbent assay (ELISA) kits following the instructions (Sdc-1; Cat #: MBS745791, MyBiosource Inc., San Diego, CA, IL-6; Cat #: SEA079Ov, Cloud-Clone Corp., Katy, TX), and as previously described^29^.

### Statistical analysis

All statistical analysis was performed using GraphPad Prism version 8.3.1 (GraphPad Software, Inc., La Jolla, CA). Results were compared between the groups at each timepoint by a two-way analysis of variance with a mixed-effects model with post hoc Bonferroni multiple comparison tests. The values measured at a single timepoint were compared by unpaired t-test or Mann–Whitney U test, based on the normality of the data distribution (Shapiro–Wilk test). All values are expressed as Mean ± standard error of mean (Mean ± SEM). Statistical significance was considered for p value < 0.05.

## Supplementary Information

Supplementary Information.

## Abbreviations

ICU: Intensive care unit
O&NS: Oxidative/nitrosative stress
NO: Nitric oxide
O2-: Superoxide anion
IACUC: Institutional animal care and use committee
TV: Tidal volume
RR: Respiratory rate
FiO2: Inspired oxygen concentration
PA: *Pseudomonas aeruginosa*
IV: Intravenous injection
IM: Intramuscular injection
BW: Body weight
LR: Lactated Ringer’s solution
qSOFA: Quick Sequential Organ Failure Assessment
mSOFA: Modified sheep Sequential Organ Failure Assessment
MAP: Mean arterial pressure
W/D: Wet-to-dry weight ratio
VEGF-A: Vascular endothelial growth factor-A
Sdc-1: Syndecan-1
IL-6: Interleukin-6
ELISA: Enzyme-linked immunosorbent assay
BT: Body temperature
HR: Heart rate
mPAP: Mean pulmonary artery pressure
CVP: Central venous pressure
SVRI: Systemic vascular resistance index
PVRI: Pulmonary vascular resistance index
CI: Cardiac index
EtCO2: End-tidal CO2
VCO2: CO2 production
LIS: Lung injury score
