# Supplementary material for: Modulation of oxidative and nitrosative stress attenuates microvascular hyperpermeability in ovine model of Pseudomonas aeruginosa sepsis
Source: Sci Rep. 2021 Dec 14;11:23966. doi: 10.1038/s41598-021-03320-w (PMC8671546; doi:10.1038/s41598-021-03320-w)
Supplement: Supplementary file 1 — Supplementary Information. [file 41598_2021_3320_MOESM1_ESM.pdf]

Title Page

Modulation of Oxidative and Nitrosative Stress Attenuates Microvascular Hyperpermeability in Ovine Model of Pseudomonas Aeruginosa Sepsis

Satoshi Fukuda[1,2], Yosuke Niimi[1,3], Yasutaka Hirasawa[1,4], Ennert R. Manyeza1, C. Edwin Garner[5], Garry Southan[5], Andrew L. Salzman[5], Donald S. Prough[1], and Perenlei Enkhbaatar[1],*

[1] Department of Anesthesiology, University of Texas Medical Branch, Galveston, TX. 77555. USA.

[2] Department of General Medicine, International University of Health and Welfare, Shioya hospital, Tochigi. 329-2145. JAPAN.

[3] Department of Plastic and Reconstructive Surgery, Tokyo Women's Medical University, Tokyo. 162-8666. JAPAN.

[4] Department of Respirology, Graduate School of Medicine, Chiba University, Chiba. 260-8677. JAPAN.

[5] Salzman Group Inc., Beverly, MA. 01915. USA

* Corresponding author: Perenlei Enkhbaatar MD, PhD, FAHA.

e-mail: peenkhba@utmb.edu Tel: +1-409-747-0096 Fax: +1-409-772-6409

Department of Anesthesiology, University of Texas Medical Branch at Galveston. 301 University Boulevard, Galveston, Texas, 77555. USA.

Supplementary Figure and Figure Legends

Supplementary Figure 1

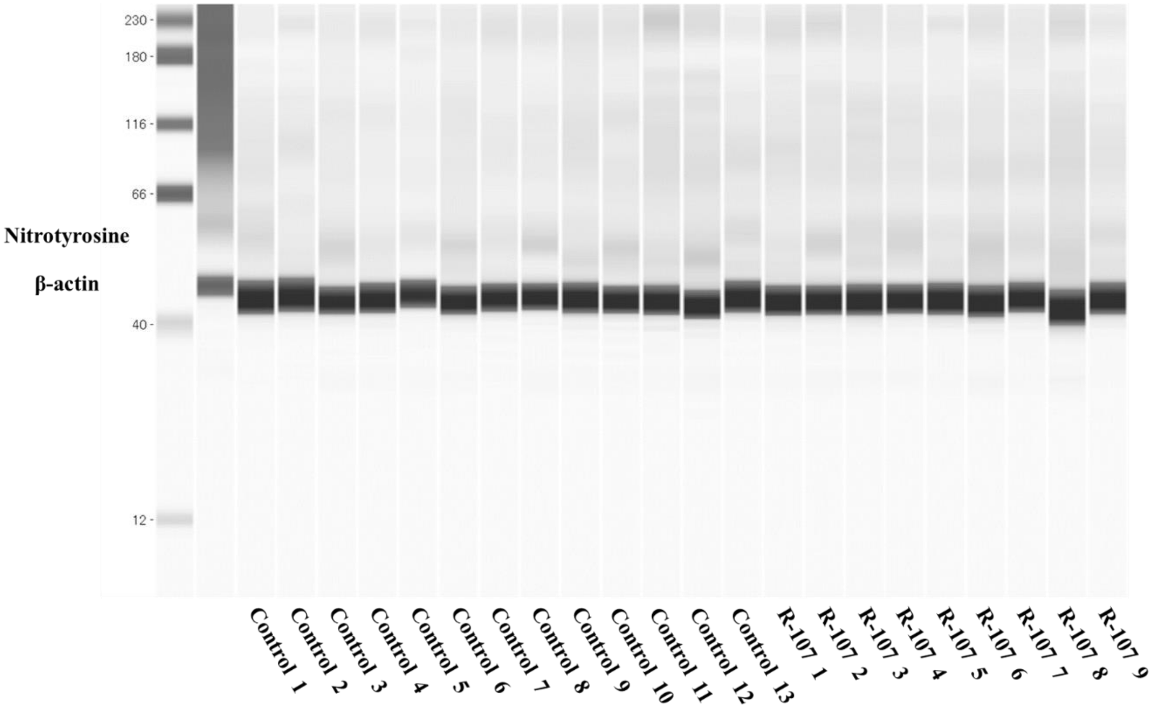

The 3-nitrotyrosine level in lung tissue homogenate collected at euthanasia in two groups were measured using anti-3-nitrotyrosine antibody (06-284; MilliporeSigma, MA) with an automated capillary Western blot analyzer (Wes, ProteinSimple, CA) system.
